# Supplementary material for: Combined BCL-2 and PI3K/AKT Pathway Inhibition in KMT2A-Rearranged Acute B-Lymphoblastic Leukemia Cells
Source: Int J Mol Sci. 2023 Jan 10;24(2):1359. doi: 10.3390/ijms24021359 (PMC9865387; doi:10.3390/ijms24021359)
Supplement: Supplementary file 1 [file ijms-24-01359-s001.zip › Supplementary Figures caption_proofread.pdf]

**Figure S1:** Evaluation of potential cytotoxic effects on non-neoplastic blood cells using IC20/30 concentrations determined in B-ALL cell lines SEM and RS4;11. Isolated PBMCs and whole blood from five healthy donors were used. **A** Inhibitors were applied as single substances and in simultaneous combination for 24 h. Subsequently PBMC viability was assessed by Calcein AM assay. Relative representation compared to control. **B** Hemolytic activity was analyzed after 120 min incubation with single and combined approaches or 1% SDS (positive control) followed by photometric detection of hemoglobin release in the cell-free supernatant.

**Figure S2:** Analysis of apoptotic processes following combined BCL-2 and PI3K/AKT inhibition. **A** Detection of activated (cleaved) caspase 3 by intracellular flow cytometry after 48 h incubation. Representation of the relative protein expression to control. Relative changes were calculated within each approach to the respective control. n≥3, ANOVA and post-hoc Tukey's multiple comparison test. * $p < 0.05$, ** $p < 0.005$, *** $p < 0.001$ **B** Immunofluorescence-based BAX translocation assay following 48 h inhibitor incubation. Representative images of at least three independent biological and technical replicates at 400x magnification.

**Figure S3:** Detection of intracellular BCL-xL and BAX expression following 48 h inhibitor incubation. Representation of the absolute protein expression/phosphorylation (left graphs), indicating the amount of target-positive cells within the entire cell population. The relative proportion of positive cells compared to control cells was calculated within each replicate and is represented in the right graphs. n≥3, ANOVA and post-hoc Tukey's multiple comparison test. * $p < 0.05$, ** $p < 0.005$, *** $p < 0.001$.

**Figure S4:** **A** Principal component analysis of the basal gene expression of four (SEM) or three (RS4;11) biological replicates. **B** Basal gene expression of main components of the BCL-2 and PI3K/AKT pathways in SEM and RS4;11 cells. Heatmap of the average total read count. Green and red color illustrate high or low reads, respectively.

**Figure S5:** Hierarchical clustering heatmap of all analyzed samples in SEM and RS4;11 cells. The heatmap represents mean log2 expression values generated by the TAC software. Red and blue color indicates high and low read counts, respectively.

**Figure S6:** Immunoblot of basal p53 protein expression. GAPDH served as loading control.

**Figure S7:** Original full-length immunoblot of p53 protein expression shown in Figure S6. Red boxes indicate cropped areas.

**Figure S8:** Effects of VEN administration on gene expression of the apoptosis signaling cascade. After 24 h inhibitor incubation, gene expression was examined by targeted RNA sequencing. Subsequent data analysis was performed with the TAC software. The WikiPathways plugin was used to integrate the fold change expression values within the apoptosis signaling cascade. Green and red color represents up- and downregulation, respectively. Genes with an average total read count <50 were excluded to justify biological significance (labelled as filtered out).

**Figures S9:** Effects of simultaneous VEN and PERI administration on gene expression of the apoptosis signaling cascade. After 24 h inhibitor incubation, gene expression was examined by targeted RNA sequencing. Subsequent data analysis was performed with the TAC software. The WikiPathways plugin was used to integrate the fold change expression values within the apoptosis signaling cascade. Green and red color represents up- and downregulation, respectively. Genes with an average total read count <50 were excluded to justify biological significance (labelled as filtered out).

**Figure S10:** Effects of combined BCL-2 and PI3K/AKT inhibition on gene expression of the extrinsic apoptosis pathway genes. VEN and PI3K/AKT inhibitors were incubated for 24 h before detection of the gene expression by targeted RNA sequencing and data analysis using the TAC software. Heatmap representation of the fold change compared to control. Green and red color represents up- and downregulation, respectively. Genes with an average total read count <50 were filtered out to justify biological significance.

**Table S1:** *BBC3* gene expression fold change values compared to controls.
