# Supplementary figures and images for: Combined BCL-2 and PI3K/AKT Pathway Inhibition in KMT2A-Rearranged Acute B-Lymphoblastic Leukemia Cells
Source: Int J Mol Sci. 2023 Jan 10;24(2):1359. doi: 10.3390/ijms24021359 (PMC9865387; doi:10.3390/ijms24021359)

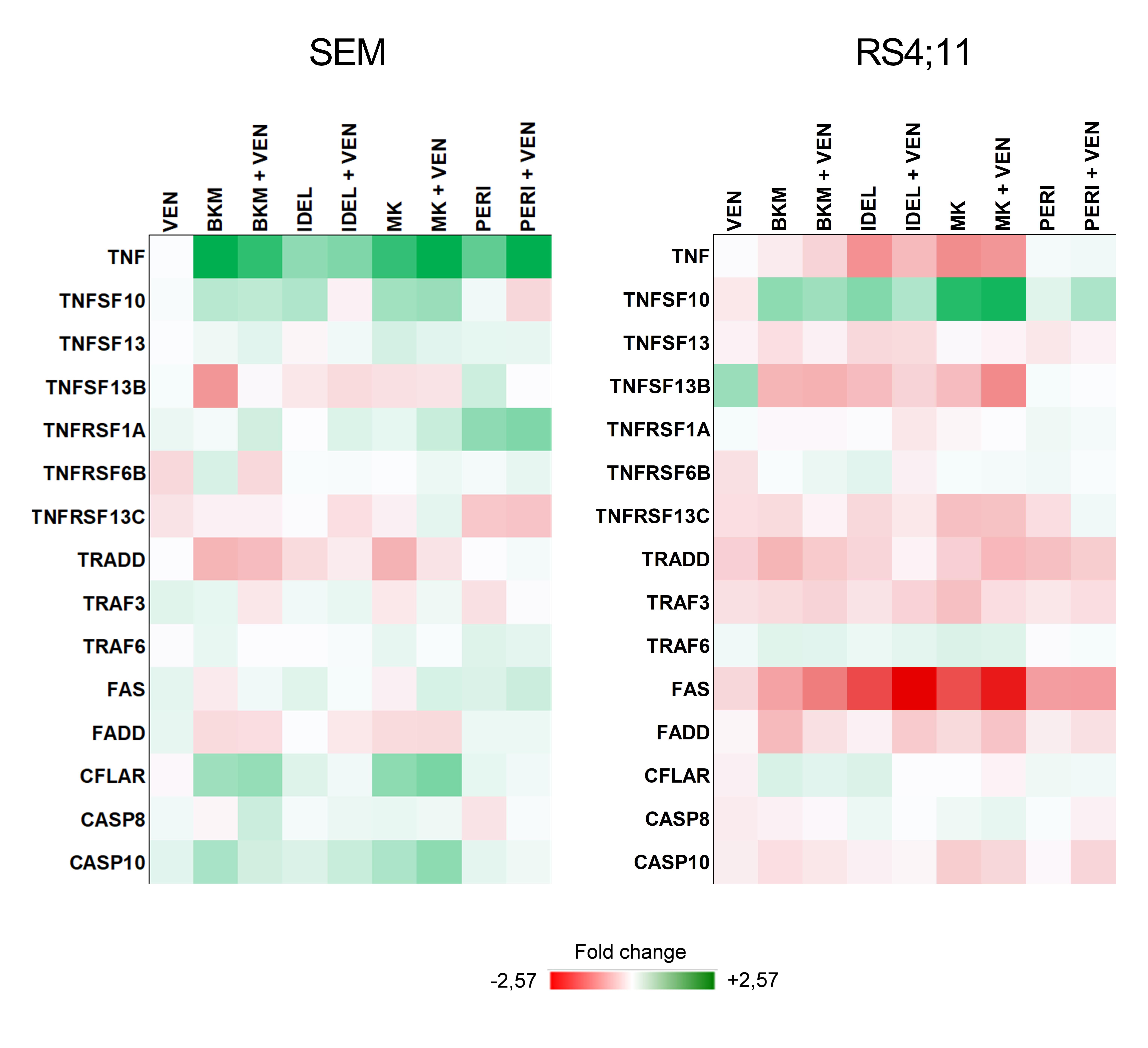

Supplement: Supplementary file 1 [file ijms-24-01359-s001.zip › Figure S10.jpg]

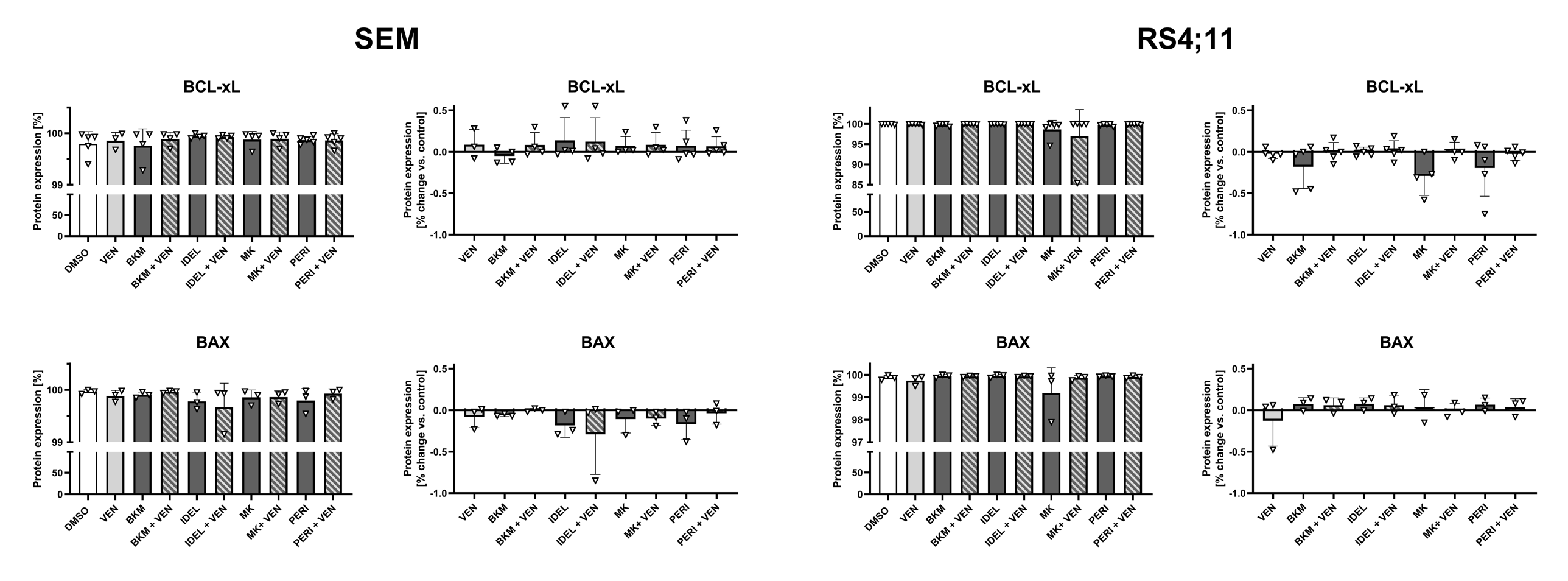

Supplement: Supplementary file 1 [file ijms-24-01359-s001.zip › Figure S3.jpg]

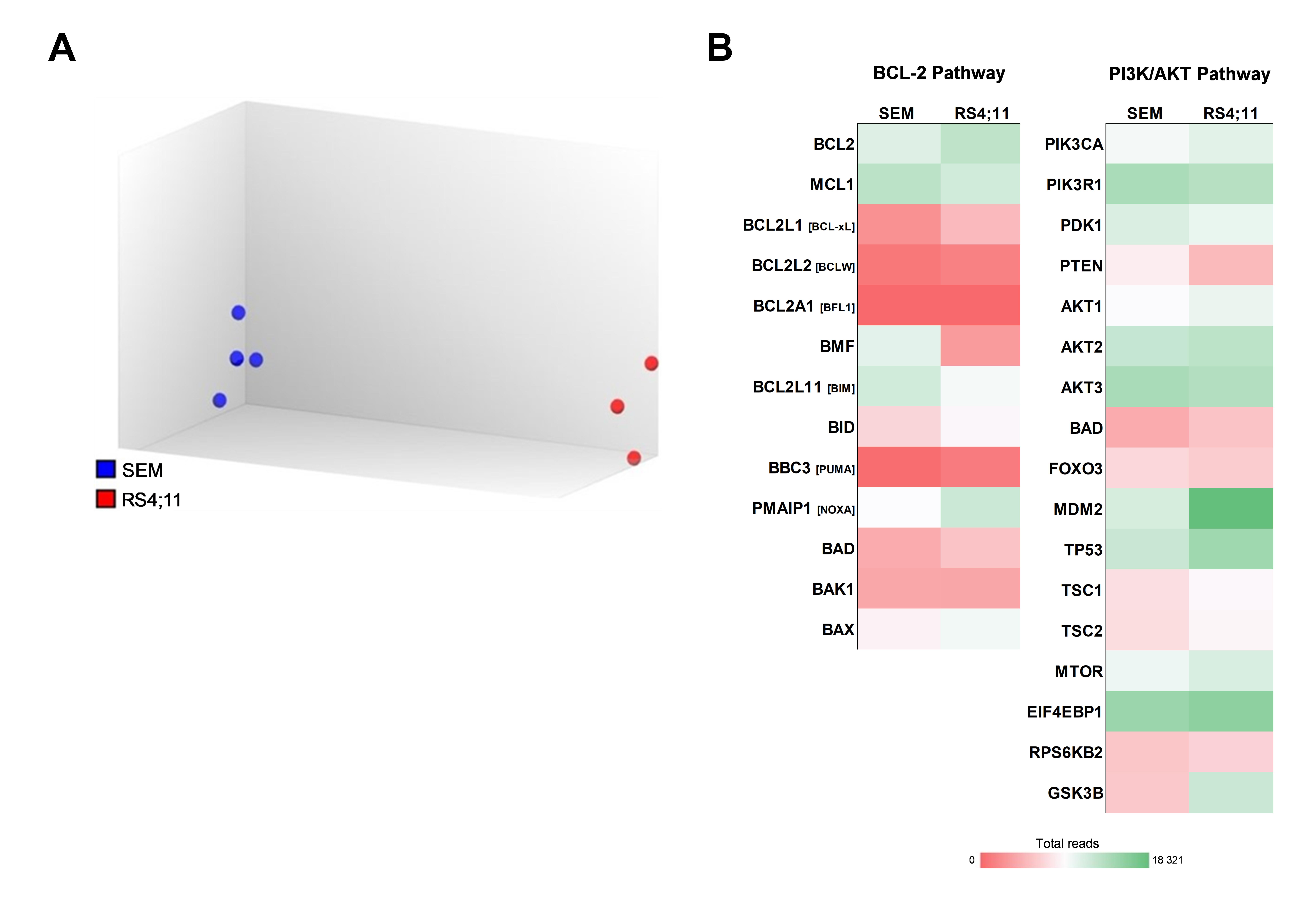

Supplement: Supplementary file 1 [file ijms-24-01359-s001.zip › Figure S4.jpg]

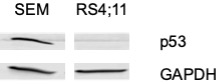

Supplement: Supplementary file 1 [file ijms-24-01359-s001.zip › Figure S6.jpg]

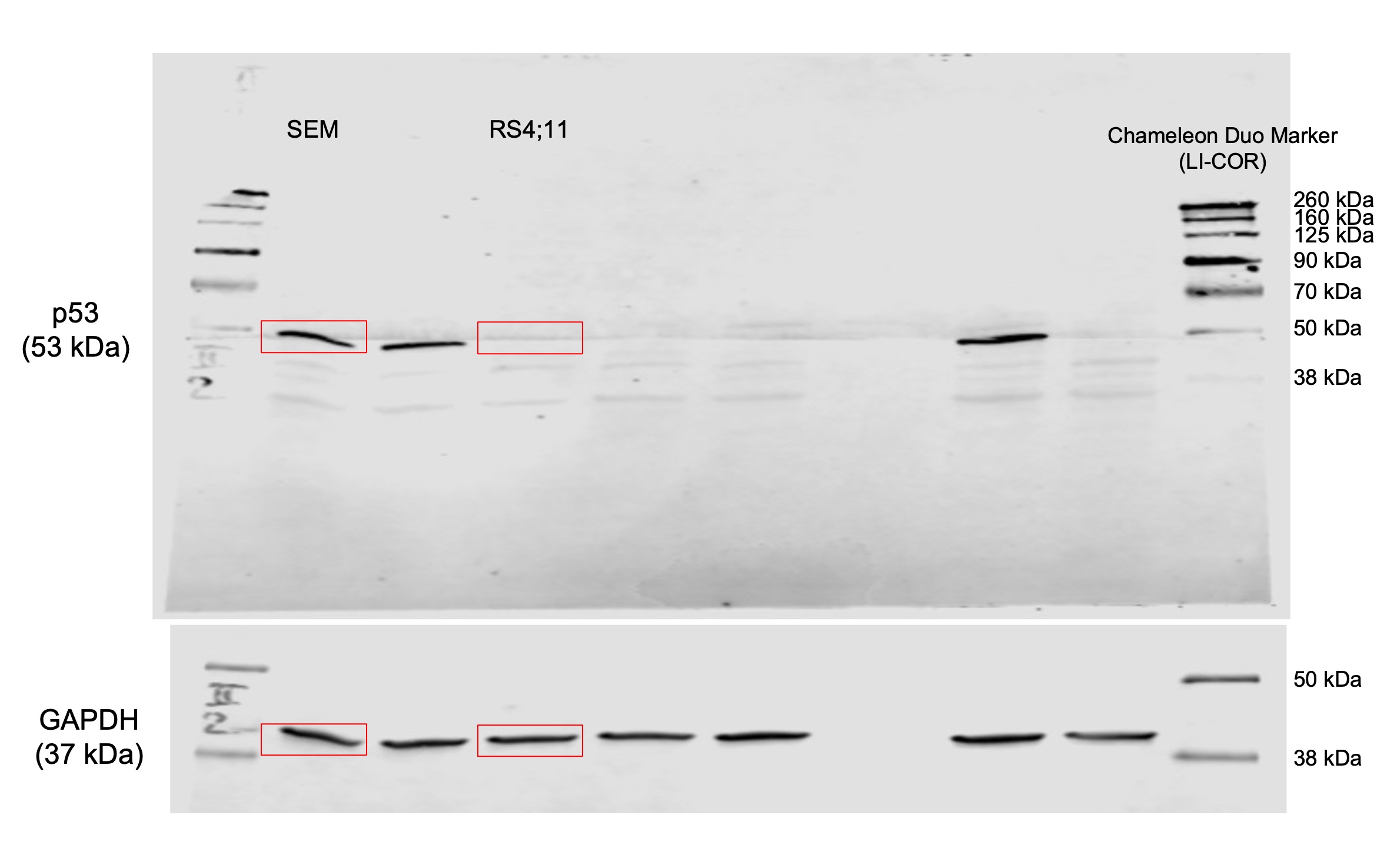

Supplement: Supplementary file 1 [file ijms-24-01359-s001.zip › Figure S7.jpg]

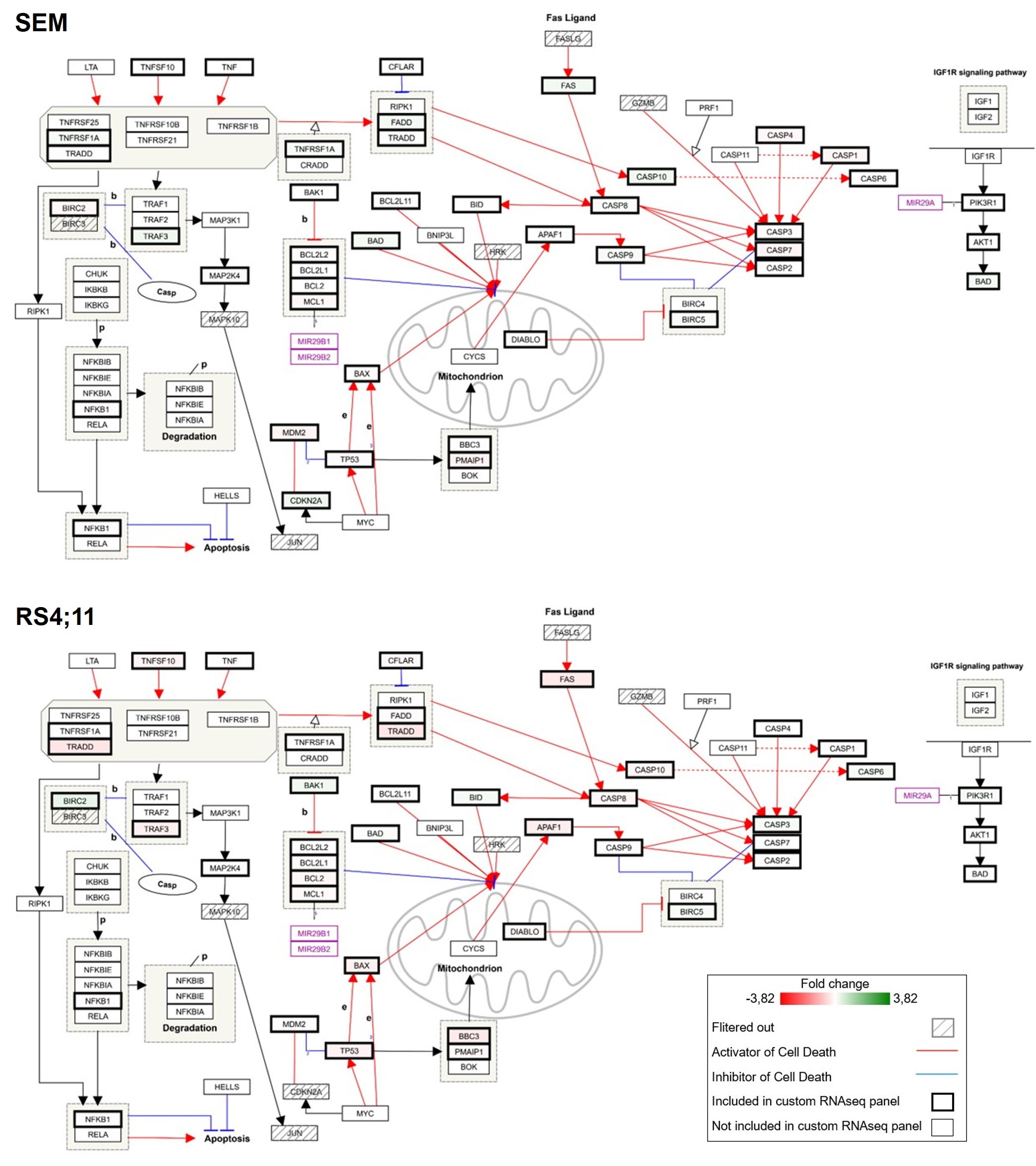

Supplement: Supplementary file 1 [file ijms-24-01359-s001.zip › Figure S8.jpg]

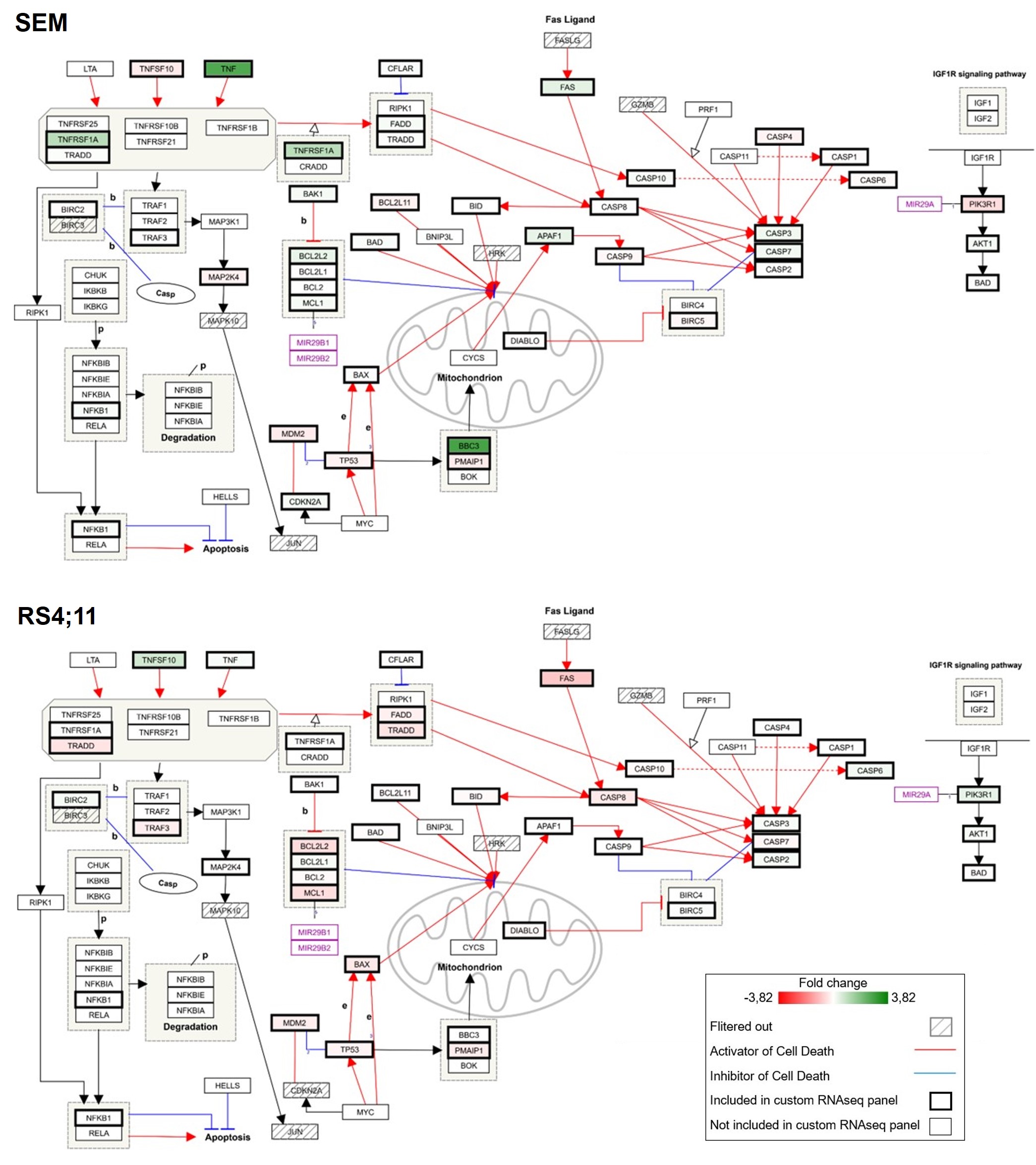

Supplement: Supplementary file 1 [file ijms-24-01359-s001.zip › Figure S9.jpg]
